# Radioiodine therapy in differentiated thyroid cancer. There is (still) a great chaos under heaven: is the situation excellent?

**DOI:** 10.1007/s12020-023-03316-8

**Published:** 2023-02-08

**Authors:** Luca Giovanella

**Affiliations:** 1 Clinic of Nuclear Medicine and Molecular Imaging, Imaging Institute of Southern Switzerland, Ente Ospedaliero Cantonale, Bellinzona, Switzerland; 2 Clinic of Nuclear Medicine, University and University Hospital of Zurich, Zurich, Switzerland

Post-operative use of Iodine-131 (^131^I) has been the standard of care in patients affected by Differentiated Thyroid Cancer (DTC) for decades. However, it has become particularly controversial since the release of the American Thyroid Association (ATA) guidelines in 2015 [1]. Most notably, the ATA low-risk and intermediate-risk classes have been expanded and high-risk class restricted compared to the ATA 2009 guidelines [2]. First, the **low-risk class** currently includes (i) clinical N0 or ≤5 pathologic N1 micrometastasis (i.e., ≤0.2 mm), (ii) intra-thyroid well-differentiated follicular thyroid carcinoma with capsular invasion and/or minimal vascular invasion (i.e., <4 foci), and (iii) intra-thyroid papillary microcarcinoma, uni- or multifocal, including BRAF V600E-mutated ones. Second, PTC patients with (i) vascular invasion, (ii) hobnail variant, (iii) clinical N1 (cN1), (iv) pathological N1 (pN1) with all lymph nodes <3 cm were downgraded from high to **intermediate risk** classes. Third, only patients with (i) macroscopic peri-thyroid invasion, (ii) incomplete tumor resection, (iii) pN1 with almost one lymph node >3 cm, (iv) follicular thyroid carcinoma (FTC) with extensive vascular invasion (>4 foci), (v) thyroglobulin (Tg) values “suggestive of distant metastases” (*whatever it means*, note of the author) remained included in the **high-risk class**.

Moreover, resulting risk classes were used to inform postoperative ^131^I therapy as follows:

- **ATA low-risk DTC patients**: remnant ablation **is not routinely recommended** after thyroidectomy (Weak recommendation, Low-quality evidence)
- **ATA intermediate-risk level DTC patients**: adjuvant therapy **should be considered** after thyroidectomy (Weak recommendation, Low-quality evidence)
- **ATA high-risk DTC patients**: adjuvant therapy is **routinely recommended** after thyroidectomy (Strong recommendation, Moderate-quality evidence).

Looking between lines, however, the situation is not so linear (admittedly some problems are elaborated in different guidelines’ chapters but, unfortunately, not considered in summary and flow-charts i.e the most read and used by clinicians, sic!).

In particular, recommendations for low- and intermediate-risk patients were scored as *weak with low-quality evidence* that implies other approaches can be equally or even more effective. Accordingly, a transparent information is required, and, beyond categorical recommendations, patients’ values and preferences should be fully considered. Moreover, a hierarchical *continuum* of additional risk factors has been proposed by the authors to “refine” the conventional risk stratification. Then, recurrence risk rates of different sub-classes were extrapolated from cohorts of patients mostly treated with postoperative ^131^I administration. Obviously, the risk estimates also reflect the effect of the radioiodine therapy in such cases while, *ca va sans dir*e, patients treated without ^131^I are likely to have different outcomes [3]. Based on relevant and animated disputes between different specialists and societies [4] the European Association of Nuclear Medicine (EANM), the Society of Nuclear Medicine and Molecular Imaging (SNMMI), the European (ETA) and the American (ATA) Thyroid Associations finally decided to discuss collegially about the use of ^131^I in DTC management, and, in the end, released a Consensus Paper [5]. As a notable starting point, panel members unanimously recognized that “*although most guidelines make recommendations based on staging systems that predict risk of recurrence or disease-specific mortality, the actual goal of* ^*131*^*I therapy can only be determined once the postoperative disease status has been assessed*”. In practical terms, recommendations based on histological risk classes alone are not enough to reach a well-balanced indication for or against postoperative ^131^I administration. Accordingly, “*patients with biochemical, structural, or functional evidence of persistent disease can only be candidates for treatment of known disease”*, regardless of initial risk stratification. On the other hand, “*patients demonstrating no histological, biochemical, or imaging evidence of persistent disease after surgery may be candidates for observation*, ^*131*^*I remnant ablation, or adjuvant* ^*131*^*I treatment”* [5]. Unfortunately, in spite of a wide diffusion and a good acceptance of the Consensus Paper little has really changed and independent guidelines and conflicting recommendations are still released by the same societies [6–9]. This is a bit disappointing when considering that “*commitment by clinicians, researchers, patients, and organizations to engage in proactive, purposeful, and inclusive interdisciplinary cooperation”* was (apparently) desired by relevant societies [5]. As an example among others, different strategies on the use of different methods to postoperatively assess DTC patients (i.e., ultrasound, thyroglobulin, whole body scan) were proposed in different guidelines with different interpretation criteria attached. Notably, reliable recommendations on postoperative assessment are not yet available and the multidisciplinary document strongly suggested to carefully take into account multiple local factors (i.e., resources, expertise, local reference values) instead of applying fixed and “universal” criteria. Just as an example, different Tg tresholds are currently proposed to inform decisions on ^131^I administration but the well-known variability between different Tg assays as well as the TSH status (*i*. measured during thyroxine therapy: suppressed or unsuppressed TSH?; *ii*. stimulated using rhTSH or thyroid hormone withdrawal?) are not considered, potentially resulting in contrasting indications simply due to methodological limitations [10, 11]. Accordingly, more efforts are required to guide colleagues in understanding technical differences and different interpretation criteria of our diagnostic methods making the role of local tumor boards crucial to avoid inappropriate decisions in clinical settings sometimes markedly different from those of most guidelines’ authors. As a matter of facts, having so many different guidelines on the stage remains incomprehensible from a logical and methodological point of view, and could be attributed more to “ideological” and “corporative” concepts rather than scientific evaluations (without exceptions among various involved actors) [4–7]. Moreover, traces of conflicting messages can be observed into the indication provided by the same institution to different stakeholders. An open-label, phase III, non-inferiority trial study randomized low-risk DTC patients to postoperative administration of 1.1 GBq ^131^I versus no ablation [12]. The authors did not find differences in outcomes after 3 years in the different groups, but acknowledged several limitations, pointing out in particular that the 3-year period applied was too short to draw definitive conclusions [13, 14]. Their critical analysis was correctly addressed to clinical readers via a well-recognized medical journal. However, a substantially different message appeared on the website of the authors’ institution (widely consulted by patients), which simply asserted that ^131^I is not useful in low-risk thyroid cancer (https://www.gustaveroussy.fr/fr/pas-de-benefice-de-liode-radioactif-dans-le-cancer-de-la-thyroide-faible-risque). In this heterogeneous context, it is clear that there is still a lot of chaos in DTC management but, *contradicting Mao Tse-Tung*, the situation is far from excellent. I myself experience almost every day how disruptive these uncertainties can be for our patients. Against this backdrop, there is an urgent need to regain scientific and clinical responsibility, respect, and collaboration and keep coherently what we say during our multidisciplinary meetings and write in our multidisciplinary papers. Developing consistent guidelines through transparent and fair inter-society cooperation is the only way to aid multidisciplinary teams in properly establishing local standards of care to guide the management of DTC patients and hopefully ameliorate the situation *under heaven* toward excellence.
